# Tumor immune microenvironment and genomic evolution in a patient with metastatic triple negative breast cancer and a complete response to atezolizumab

**DOI:** 10.1186/s40425-019-0740-8

**Published:** 2019-10-23

**Authors:** Luciana Molinero, Yijin Li, Ching-Wei Chang, Sophia Maund, Maureen Berg, Jeanne Harrison, Marcella Fassò, Carol O’Hear, Priti Hegde, Leisha A. Emens

**Affiliations:** 1 Oncology Biomarker Development, PDCO-Immunolotherapy, Genentech, Incorporated, 1 DNA Way MS: 245c, South San Francisco, CA 94080 USA; 2 School of Medicine, Oncology/Immunology, Johns Hopkins University, The Skip Viragh Outpatient Cancer Building, Floor 8, Viragh 8200-30, Box 11, 201 N Broadway, Baltimore, MD 21287 USA; 3 Bloomberg~Kimmel Institute for Cancer Immunotherapy at Johns Hopkins, Baltimore, USA; 4 University of Pittsburgh Medical Center, Hillman Cancer Center, 5117 Centre Avenue, Room 1.46e, Pittsburgh, PA 15213 USA

**Keywords:** Triple negative breast cancer (TNBC), Atezolizumab, PD-L1

## Abstract

**Background:**

Metastatic TNBC (mTNBC) has a poor prognosis and few treatment options. The anti-PD-L1 antibody atezolizumab demonstrated clinical activity in mTNBC patients with PD-L1-positive tumor-infiltrating immune cells. The current study describes the tumor immune microenvironment (TiME) and genomic evolution across sequential therapies in a patient with a 31-year history of TNBC and a complete response (CR) to atezolizumab monotherapy.

**Materials and methods:**

In 1986, the patient had surgery and radiotherapy (XRT) for newly diagnosed TNBC, followed by surgery and adjuvant chemotherapy for two locoregional recurrences. She developed mTNBC in 2009 and was sequentially treated with capecitabine, gemcitabine-carboplatin-iniparib (GCI), XRT and an experimental vaccine. She experienced disease progression (PD) to all these therapies. In 2013, she had a PD-L1 positive tumor and enrolled in a phase 1 atezolizumab monotherapy study (PCD4989g; NCT01375842). She received atezolizumab for 1 year with initial pseudo-progression followed by a partial response. After 1 year without treatment she experienced PD, reinitiated atezolizumab and subsequently achieved CR. Tumor specimens were collected at numerous times between 2008 and 2015 and assessed by immunohistochemistry, RNA-seq and DNA-seq.

**Results:**

TiME biomarkers, including CD8, ICs and PD-L1 on IC, increased after capecitabine and remained high after GCI, XRT and through pseudo-progression on atezolizumab. At PD post-atezolizumab exposure, TiME biomarkers decreased but PD-L1 status remained positive. Immune-related RNA signatures confirmed these findings. TNBC subtyping revealed evolution from luminal androgen receptor (LAR) to basal-like immune activated (BLIA). Genomic profiling showed truncal alterations in *RB1* and *TP53*, while the presence of other genomic alterations varied over time. Tumor mutational burden peaked after XRT and declined after atezolizumab exposure.

**Conclusions:**

This case report describes the evolution of TiME and TNBC molecular subtypes/genomics over time with sequential therapies in a TNBC patient with a CR to atezolizumab monotherapy. These data suggest the TiME is pliable and may be manipulated to maximize response to immunotherapy (NCT01375842, https://clinicaltrials.gov/ct2/show/NCT01375842?term=NCT01375842&rank=1).

## Background

The main therapy for metastatic triple negative breast cancer (mTNBC) in the United States (US) is cytotoxic chemotherapy [1]. mTNBC has a worse prognosis than other breast cancer subtypes, with a median overall survival (OS) variously reported in the literature of 8–13 months [2]. Chemotherapy remains the cornerstone of therapy for TNBC, though bevacizumab and olaparib may be used in selected settings. Recently, atezolizumab plus nab-paclitaxel demonstrated clinical benefit in patients with PD-L1+ tumors in the clinical study IMpassion130 (NCT02425891) [3].

TNBC is more likely to harbor tumor-infiltrating lymphocytes (TILs) than other breast cancer subtypes [4]. Higher levels of TILs at diagnosis are associated with a favorable prognosis to standard therapies in early TNBC [5]. TNBC is also more likely than other breast cancer subtypes to express PD-L1, a predictive biomarker for atezolizumab clinical benefit [3, 6, 7]. Blockade of single agent PD-1/PD-L1 pathway results in durable clinical responses across a range of tumor types, with response rates in solid tumors averaging 10–30% [8].

The humanized anti-PD-L1 antibody atezolizumab inhibits the interaction of PD-L1 with the receptor PD-1, enabling the reactivation of dysfunctional T cells [9]. In the clinical study PCD4989g (NCT01375842), atezolizumab monotherapy has demonstrated a response rate of 10–13% of mTNBC, where responses are associated with PD-L1 expression in immune cells (IC) as well as increased IC levels [7]. A deeper understanding of the biomarkers linked to clinical response in TNBC may enable rational patient selection and facilitate an informed use of atezolizumab for TNBC patients in the clinic. This study describes the immunogenomic evolution of TNBC across sequential therapies in a remarkable patient with a 31-year history of TNBC and a complete response (CR) to atezolizumab monotherapy.

## Materials and methods

### Peripheral blood biomarkers

Absolute lymphocyte counts (cells/μL) (CD3+, CD3 +CD4+, CD3+CD8+), B cells (CD19+) and NK cells (CD56+/CD16+) and percentages of CD8+/HLA-DR+/KI67+ T cells were determined from heparinized whole blood using standard flow cytometry methods. IL-18, CXCL10, GZMA, CEA and CA27–29 were analyzed in plasma using luminex (Myriad-RBM) and ELISA assays.

### Histopathological assessments

Tumor-infiltrating ICs (lymphocytes, macrophages, dendritic cells and granulocytes) as detected by hematoxylin and eosin (H&E) staining were scored as a percentage of the tumor area comprised of tumor cells and desmoplastic stroma [7]. PD-L1 expression on immune cells and tumor cells was evaluated using the VENTANA SP142 immunohistochemistry (IHC) assay (Ventana Medical Systems, Tucson, AZ, USA) [9]. Immunohistochemistry was centrally performed (HistoGeneX, Antwerp, Belgium) using C8/144B (CD8) and MRQ-26 (CD163) antibody clones (Dako, Glostrup, Denmark). Aggregated data for these biomarkers from the TNBC cohort in the PCD4989g study [7] is provided in Additional file 1: Table S1.

### RNA and DNA sequencing assessments

Gene expression levels were quantified by TruSeq RNA Access RNA sequencing (Illumina, CA, USA) [10, 11]. For comparison, the data for the individual tissue samples are displayed along the aggregated values from the rest of the PCD4989g TNBC cohort (Table S1, *n* = 103) [12]. TNBC molecular subtypes were assigned as previously described [13], with modifications to adapt for the use of formalin-fixed, paraffin-embedded tissues obtained on our study. Mutation detection, tumor mutational burden, somatic/germline status and clonality were assessed using the FoundationOne® platform as previously described (Foundation Medicine, Inc., Cambridge, MA, USA) [14–16].

## Results

### Case presentation

**Fig. 1:**
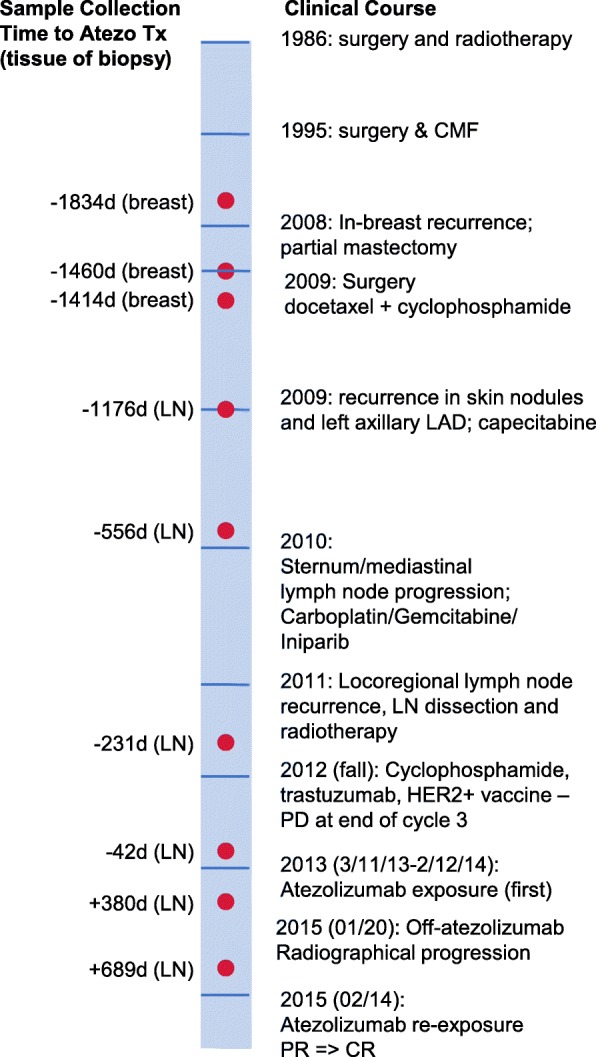
Clinical course of disease and time-points of collected tumor biopsies. On the right: chronological schema of disease appearance and treatments, on years. On the left: time of sample collection (red dots) on days respect to initiation to atezolizumab first exposure. CMF: cyclophosphamide, methotrexate and fluorouracil; Biopsy Dx: diagnostic biopsy; LAD: lymphadenopathy

**Fig. 2:**
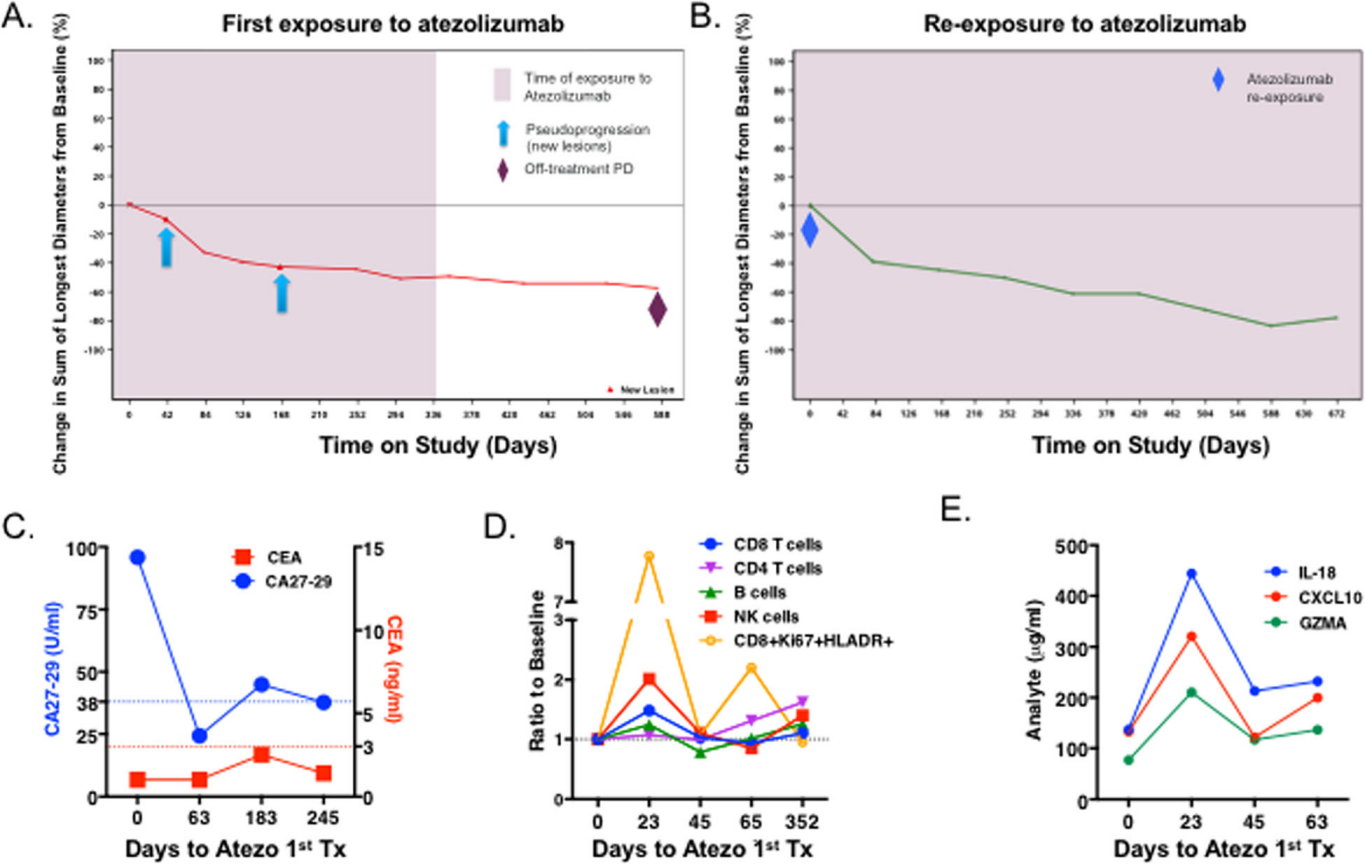
Change in tumor burden and circulating biomarkers after atezolizumab exposures. Changes in sum of lesion diameter (SLD) was assessed over time in initial (**a**) and second (**b**) exposure to atezolizumab. Plasma tumor antigens CEA and CA27–29 (**c**), circulating T, B and NK lymphocytes (**d**) and the cytokines IL18, CXCL10 and granzyme A (GZMA)) (**e**) were assessed over time during the first exposure to atezolizumab. Upper limit of normal levels for CEA (3 ng/ml) and CA27–29 (38 U/ml) are indicated by the dotted lines (blue: CA27-29; red: CEA). Changes in lymphocyte populations were plotted as ratio to baseline to pre-treatment values

A 48-year-old woman with a long history of TNBC (Fig. 1) was enrolled in the Phase 1a study of single agent atezolizumab (PCD4989g; NCT01375842) on March 5, 2013. In 1986, she presented with an early right breast cancer negative for the estrogen and progesterone receptors. Initial management included lumpectomy and radiotherapy, and then two chest wall recurrences were treated with surgery and adjuvant chemotherapy (1993). In 2009, the patient presented with another early right TNBC treated with surgery followed by chemotherapy with docetaxel + cyclophosphamide (T + C). She then developed skin nodules and left axillary lymphadenopathy and received palliative capecitabine. In 2010, she developed progressive mTNBC involving the sternum and mediastinal lymph nodes and received 9 cycles of gemcitabine, carboplatin, and iniparib (GCI) until summer 2011. In late 2011, lymph node recurrence was treated with axillary lymph node dissection (2/5 lymph nodes involved with tumor) and radiotherapy. In 2012, she developed progressive disease (PD) and received 3 cycles of a whole cell breast tumor vaccine, trastuzumab, and low dose cyclophosphamide (NCT00971737). Upon progression, she was enrolled in the Phase 1a PCD4989g atezolizumab monotherapy trial, first dosed on March 11, 2013. She had a partial response (PR) by Response evaluation criteria in solid tumors (RECIST) and Immune-related Response Criteria (irRC) after 4 cycles. On May 31, 2013, the patient had an irPR per irRC and unconfirmed PR per RECISTv1.1. In July 2013 she experienced a pseudoprogression: appearance of a new nodal lesion (PD by RECISTv1.1) while still responding according to irRC. She was clinically well and continued treatment. On November 2013, the patient progressed by both RECISTv1.1 and irRC with the appearance of two new nodal lesions. On January, 2014, her target lesions were still in response (Fig. 2a), while the new nodal lesions were enlarged but stable. On February 2014, after 16 cycles, she discontinued atezolizumab exposure per protocol, with close surveillance.

On March 26, 2014, she was diagnosed with a catheter-related deep vein thrombosis, with left arm edema and enlarged lymph nodes; lymph node biopsy showed scattered tumor cells, while she had an ongoing PR in the target lesions. On July 2014, she developed palpable left axillary lymph nodes with poorly differentiated carcinoma with extensive necrosis (Fig. 2b). On January 2015 a CT scan showed nodal PD and she re-initiated single agent atezolizumab on February 2015. While on re-treatment with atezolizumab she first had a PR in April 2015, and then a complete response (CR) in June 2016. As of the clinical data cutoff (December 31, 2016), she remained on treatment and in CR (Fig. 2). This patient provides a unique opportunity to profile the immunogenomic evolution of the TiME before and during atezolizumab therapy.

### Transient changes in peripheral lymphocytes and cytokines

The impact of atezolizumab on surrogate plasma biomarkers of breast cancer progression (CA27–29 and CEA) and T-cell activation was evaluated. Plasma CA27–29 decreased prior to the first radiologic response (+63d) but increased prior to PD (+183d) and then normalized (+245d, Fig. 2c), suggesting a progression between two response episodes. Consistent with a systemic pharmacodynamic biomarker effect of atezolizumab [9], CD8 + Ki67 + HLA-DR+ cells increased transiently after one cycle of atezolizumab (23d, 7.8-fold), followed by a nadir, then a slow rise over one year of treatment (Fig. 2d). Natural killer (NK) cells and CD8+ T cells followed a similar pattern, but the change was less profound (2- and 1.4-fold on +23d, respectively) (Fig. 2d). Similarly, interleukin-18 (IL-18) and CXCL10 (cytokines/chemokines induced by IFNγ), and extracellular granzyme A (GZMA, produced by cytotoxic T lymphocytes and NK cells) peaked after one cycle of atezolizumab and then returned to baseline (Fig. 2e). Although these changes in peripheral blood biomarkers over time have not been associated with atezolizumab clinical activity, they indicate a systemic but transient atezolizumab-induced systemic T cell activation [9].

### Evolution of the tumor immune microenvironment (TiME)

**Fig. 3:**
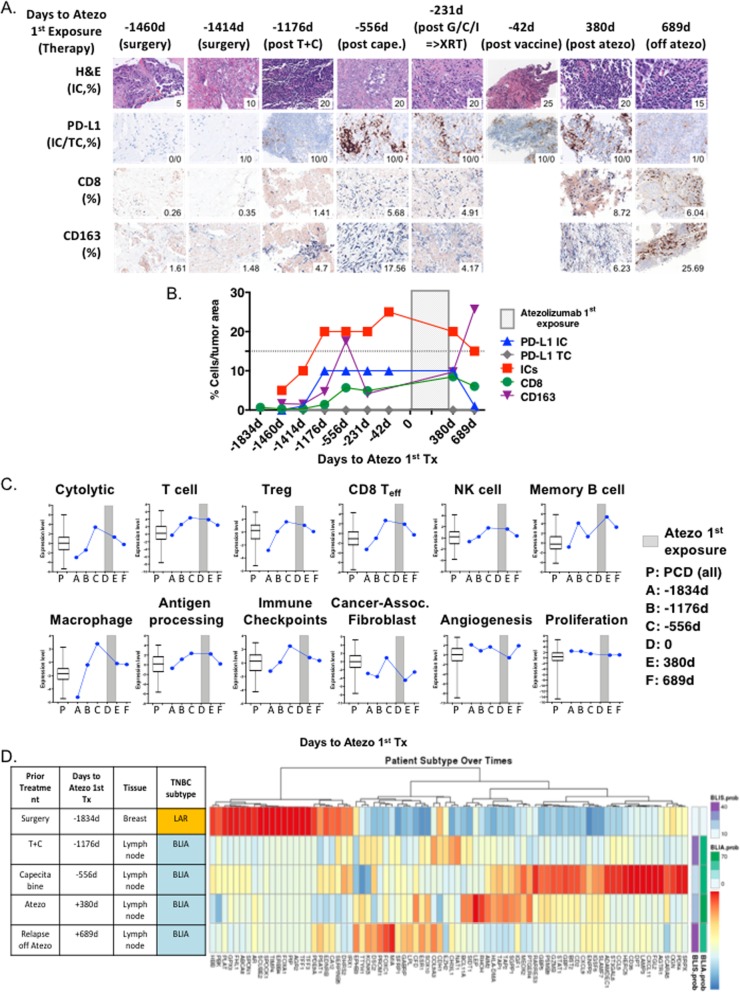
Evolution of Tumor Microenvironment. Samples collected over time. Images at 100X (**a**) of tumor infiltrating immune cells (ICs), PDL1 in tumor cells and tumor infiltrating immune cells, CD8 T cells and CD163 macrophages were assessed by hematoxylin& eosin or immunostaining. **b** Quantification of the parameters in (A) are displayed as a percentage of tumor area and evaluated over time with respect to atezolizumab first exposure. **c** RNA-based signatures associated with T cells, regulatory T cells, CD8 effector T cells, NK cells, B cells, macrophages, immune checkpoints, cancer associated fibroblasts, cytolytic activity, antigen processing, angiogenesis, and proliferation were derived from RNA-Seq and plotted as PC1 scores and displayed over time. As reference, the aggregated value for the samples from the TNBC cohort in the PCD4989g study (PCD, all) is displayed as box plots representing median, 25th and 75th percentiles and the vertical bars represent range (maximum and minimum). **d** TNBC subtype classifiers were derived from RNA-Seq for each sample. Heatmap denotes relative RNA expression of genes involved in the subtype classifiers in the analyzed samples. BLIA and BLIS prob.: probability that the samples are BLIA or BLIS

Multiple tissue biopsies were collected over the patient’s clinical course, providing an opportunity to evaluate temporal changes in the TiME after successive therapies. PD-L1 expression on ICs and tumor cells, ICs by H&E, CD8+ T cells, and CD163+ macrophages was evaluated using IHC. Additional file 1: Table S1 serves as reference of for the median, range and interquartile values for the totality of the samples collected in the TNBC cohort of the PCD4989g clinical study [7]. Immune infiltration (ICs and CD8+ T cells) and PD-L1 IC expression were low in the early disease tumor specimens and increased after T + C and capecitabine exposure (−1176d to -556d, Fig. 3a and b). Over the course of further chemo/radiotherapy and breast tumor vaccine, ICs, CD8+ T cells, and PD-L1 IC expression remained elevated. Throughout atezolizumab exposure (+380d), ICs, CD8+ T cells, and PD-L1 IC expression were high, but decreased at relapse off atezo first exposure (+689d), most significantly for PD-L1 IC (10 to 1%) and to a lesser extent ICs (20 to 15%) and CD8s (8.5 to 6%). CD163+ M2 macrophages, usually associated with immunosuppression, first increased post-capecitabine, further increased post-GCI and XRT, declined after the tumor vaccine immunotherapy, remained low through the suspected pseudo-progression on atezolizumab (+380d) and peaked again at relapse off-atezo. The tumor had elevated immune infiltration and PD-L1 IC expression and peaked at relapse off-atezo (+689d, 6.23 to 25.69%). PD-L1 in the tumor cells was never detected (Fig. 3b). These results suggest that while progression while off atezolizumab had reduced PD-L1 IC, the tumor sample was still PD-L1 IC+ (≥1%) [3, 7].

### RNA Seq-based gene expression analyses

To evaluate the evolution of the immune, stromal and tumor biology, RNA-based gene expression signatures were analyzed in a subset of tumor samples with evaluable material (−1834d, −1176d, −556d, +380d and + 689d) and compared to the aggregated levels for all the TNBC patients in the PCD4989g study (Fig. 3c). Consistent with the IHC findings, RNA-signatures associated with T cells, NK cells, antigen presentation, cytolytic capacity and immune checkpoints were low in the primary tumor and increased over time, prior to and while on atezolizumab. All of these signatures declined, except for macrophages, at the time of PD while off atezolizumab first exposure (+689d, Fig. 3c). Tumor stroma and angiogenesis have been associated with poor clinical outcomes in early TNBC [17, 18]. RNA-based signatures for cancer-associated fibroblasts (CAFs) were overall low, except of an increase post-capecitabine (−556d), but returned to lower levels post-atezo. Angiogenesis and proliferation gene signatures were overall medium-high throughout the clinical course of the patients, regardless of therapy. The RNA-based results support the immune IHC data and that off atezo relapse is not associated to overall loss of TiME.

### Temporal plasticity of TNBC subtypes

TNBC is a heterogeneous disease composed of several molecular subtypes. Four distinct TNBC subtypes were identified by RNA profiling: luminal androgen receptor (LAR), mesenchymal (MES), basal-like immune-suppressed (BLIS), and basal-like immune-activated (BLIA) [13]. In early TNBC the prognosis is worst for BLIS tumors and favorable for BLIA tumors. TNBC subtype profiling of the tumor samples showed an evolution from LAR in the primary tissue (−1834d) to BLIA in the lymph node metastasis after TC (−1176d, −556d, +380d, +689d) (Fig. 3d). Post-TC (−1414d) and at PD off-atezolizumab (+689d), the BLIA samples had a significant BLIS component (38 and 42%, respectively), while the post-capecitabine (07/02/2011) and post-atezolizumab exposure (+380d) samples had a lower BLIS component (17 and 26%, respectively) (Fig. 3d**)**. These fluctuations in LAR/BLIA/BLIS biology seemed consistent with the immunohistological and RNA variations described above.

### Characterization of the genomic landscape over time

**Fig. 4:**
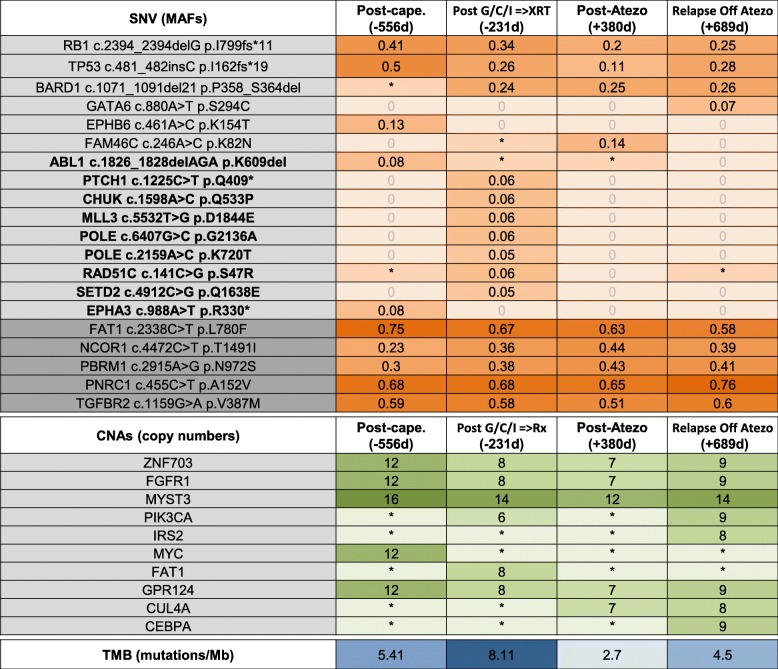
Characterization of Genomic Landscape Over Time. Samples collected pre- and post-atezolizumab exposure were tested with the FoundationOne® targeted NGS assay. Upper panel: genes with detected single nucleotide variants (SNV). Mutant allele frequencies (MAF) are shown for each specimen. Asterisk (*) indicates that the variant was present at a frequency below the validated reporting threshold. Light gray: predicted somatic mutations, dark gray: predicted germline mutations; Bold: predicted subclonal somatic mutations. Middle panel: genes with detected copy number alterations (CNAs). Numbers indicate the number of copies detected. Asterisk (*) indicates that low-level amplifications were detected below the validated reporting threshold of > 5 copies. No homozygous deletions were observed. Lower panel: tumor mutational burden (TMB) indicated as mutations per megabase

Studies in bladder cancer, lung cancer and melanoma have shown an association between high tumor mutational burden (TMB) and response to immune checkpoint blockade [19, 20]. This patient’s tumor biopsies were subjected to comprehensive hybrid-capture-based genomic profiling (FoundationOne® assay) (Fig. 4). 23 genes were identified with single nucleotide variants (SNVs), 7 of which were truncal and present in all tumor samples obtained pre- and post-atezolizumab (−556d, −231d, +380d, +689d). Truncal copy number alterations (CNAs) of ZNF703, FGFR1, MYST3 and GPR124 were detected in all samples, while frequencies of CNAs in PIK3CA, IRS2, MYC, FAT1, CUL4A, MYC and CEBPA were less consistent but were present in all samples below the assay’s validated reporting threshold. Deleterious mutations in the tumor suppressors *TP53* and *RB1* emerged as possible oncogenic driver mutations, with amplification of the epigenetic activator MYST3 as a possible modifier. Subclonal somatic SNV mutations peaked post-XRT and were not detected after atezolizumab exposure. This observation is consistent with the possibility that radiation generated neoantigens targeted by anti-tumor T cells, further activated by atezolizumab. Similarly, the TMB was highest post-XRT (TMB = 8.11 Mut/Mb, −231d), and lowest post-atezolizumab (TMB = 2.7 Mut/Mb, +380d, reference in Additional file 1: Table S1). The temporal evolution of the tumor genomic landscape (SNV and TMB) suggests that low frequency clones appear during cancer therapies. It is possible that atezolizumab might have activated T-cells targeted against immunogenic tumor cell clones.

## Conclusions

The TNBC TiME of a singular patient with very long course of TNBC was evaluated by IHC and genomic profiling in multiple tumor biopsies collected over the course of several therapies. Four findings relevant to immunotherapy for mTNBC are reported: (1) the TiME is dynamic and may evolve over time under the influence of standard cancer therapies or other environmental factors, (2) the TNBC subtype may also evolve, (3) the tumor mutational burden may change, and (4) truncal somatic mutations may persist while subclonal mutations vary upon exposure to therapies.

This patient is unusual, with a long history of TNBC that spans over 30 years, of which she bore seven with metastatic disease, much longer than the 12 months of survival for most metastatic patients with TNBC [2]. Her disease was initially managed with locoregional therapy (excision and radiation therapy), then systemic chemotherapy, and ultimately immunotherapy. She is also unusual in that she had an atypical response to atezolizumab, as this patient experienced a pseudoprogression (PD by RECIST v1.1/PR by irRC) followed by an unequivocal response by RECIST v1.1 and irRC. Three weeks after the first dose, she developed transient and mild activation of the immune system as reflected by increased numbers of proliferating CD8+ T cells and NK cells and higher levels of the interferon-γ-related cytokines IL-18 and CXCL10, consistent with the pharmacodynamic effects of atezolizumab [9]. Shortly thereafter she was noted to have a PR in the setting of declining tumor burden markers. She went on to experience pseudo-progression, with an increase in nodal disease but ongoing clinical benefit. She continued therapy and per protocol she discontinued atezolizumab after one year, with close follow-up. After one year off therapy she developed unequivocal progressive disease, and atezolizumab was re-introduced. She went on to develop a durable complete response, which persists today. It is possible that the drug holiday allowed the tumor clones susceptible to atezolizumab to regrow and outcompete the resistant ones, hence the second round of treatment was effective because it was targeting the CIT-susceptible tumor clones.

The TNBC subtypes, LAR, MES, BLIS, and BLIA, defined by RNA profiling subtypes have distinct prognoses in early TNBC [13]. Prognosis by subtype in the early setting suggest that BLIS and BLIA are the worst and best prognosticators, while the MES and LAR are intermediate. In the PCD4989g study, patients with mTNBC whose tumors were BLIA, but not LAR or BLIS, had the highest response and longest overall survival to atezolizumab monotherapy [12]. The patient in the current report had a LAR subtype in her original breast tumor, which may have been less aggressive than BLIS and MES subtypes, and upon capecitabine exposure her tumor evolved into BLIA, which carries a better prognosis. RNA immune signatures are informative clinical predictors in ER-negative early breast cancers [21] and to atezolizumab monotherapy [12].

While emerging data points to a reduction of immune components in metastases vs primary tumors, our report describes that a less infiltrated tumor may become inflamed with subsequent therapies. Preclinical and clinical studies have indicated that chemotherapy and radiotherapy may prime the TiME to immune checkpoint inhibitors [22]. Emerging data points to short term exposure to doxorubicin and platinum as boosters for nivolumab activity in TNBC [23]. Although the patient in the current case report had received various therapies prior to atezolizumab exposure, it is unclear which of them (or their combination) primed the TiME to respond to atezolizumab monotherapy.

Greater number of subclonal single nucleotide variants (SNVs) were detected post-XRT and fewer after atezolizumab exposure. While sampling bias cannot be ruled out, the observation that somatic tumor mutation subclones are eliminated upon atezolizumab exposure is consistent with the hypothesis that tumor subclones are immunogenic and susceptible to T cell-mediated killing. Subclonal neoantigens are targets of the immune response elicited by blockade of the PD-1 axis [24], and neoantigen loss through the elimination of tumor subclones or through deletion of chromosomal regions that contain truncal mutations can result in resistance to immune checkpoint blockade [25].

Mechanisms of acquired resistance to checkpoint inhibitors include loss of IFNγ-transducing signaling pathways JAK1 and JAK2, loss of antigen presentation (B2M) and activation of the PTEN/PI3K pathway in pembrolizumab-treated melanoma patients [26]. No mutations in these pathways were observed in our case report patient at the time progression while off atezolizumab. On the other hand, immunosuppressive CD163 M2 macrophages peaked at time of progression. Still, pre-treatment levels of CD163 M2 macrophages were not linked to lack of atezolizumab clinical activity in mTNBC patients treated with atezolizumab monotherapy [7], suggesting that this change in the microenvironment may not have been associated to lack of atezolizumab activity.

TNBC is a heterogeneous disease that presents a major therapeutic challenge compared to targeted therapies for luminal (estrogen and progesterone receptors) and HER-2+ breast cancers. Atezolizumab has demonstrated promising clinical activity in mTNBC proof of concept studies, with response rates of 10% for single agent and 40% in combination with nab-paclitaxel [7, 27]. The confirmatory phase 3 clinical study IMpassion130 further demonstrated that atezolizumab + nab-paclitaxel patients bearing PD-L1 IC+ (≥1%) tumors derived a clinically meaningful benefit whereas those patients with PD-L1 IC- (< 1%) tumors did not [3, 28]. Furthermore, the IMpassion130 study showed that presence of TILs and CD8 T cells in PD-L1 IC- tumors were not associated to atezolizumab plus nab-paclitaxel clinical benefit [28]. Notably, the PD-L1 status in this patient was positive prior to both atezolizumab exposures, which supports the response to atezolizumab in both instances. Characterizing the tumor microenvironment of robust responders should provide additional insights into the most informative biomarkers of clinical benefit to immune checkpoint inhibitors in mTNBC.

## Supplementary information

**Additional file 1: Table S1.** Median, interquartiles and range aggregated values for PD-L1 IC, PD-L1 TC, ICs, CD8, CD163 and TMB in TNBC samples from patients enrolled in the PCD4989g clinical study. Values represent the totality of all pre-treatment and post-treatment analyzed samples.

## Data Availability

Not applicable.

## Abbreviations

CAFs: Cancer-associated fibroblasts
CNAs: Copy number alterations
CR: Complete response
H&E: Hematoxylin and eosin
ICs: Immune cells
irRC: Immune-related Response Criteria
RECIST: Response evaluation criteria in solid tumors
TILs: Tumor-infiltrating lymphocytes
TiME: Tumor immune microenvironment
TMB: Tumor mutational burden
TNBC: Triple negative breast cancer
