# Supplementary material for: Tumor immune microenvironment and genomic evolution in a patient with metastatic triple negative breast cancer and a complete response to atezolizumab
Source: J Immunother Cancer. 2019 Oct 23;7:274. doi: 10.1186/s40425-019-0740-8 (PMC6813065; doi:10.1186/s40425-019-0740-8)
Supplement: Supplementary file 1 — Additional file 1: Table S1. Median, interquartiles and range aggregated values for PD-L1 IC, PD-L1 TC, ICs, CD8, CD163 and TMB in TNBC samples from patients enrolled in the PCD4989g clinical study. Values represent the totality of all pre-treatment and post-treatment analyzed samples. [file 40425_2019_740_MOESM1_ESM.pptx]

## Slide 1
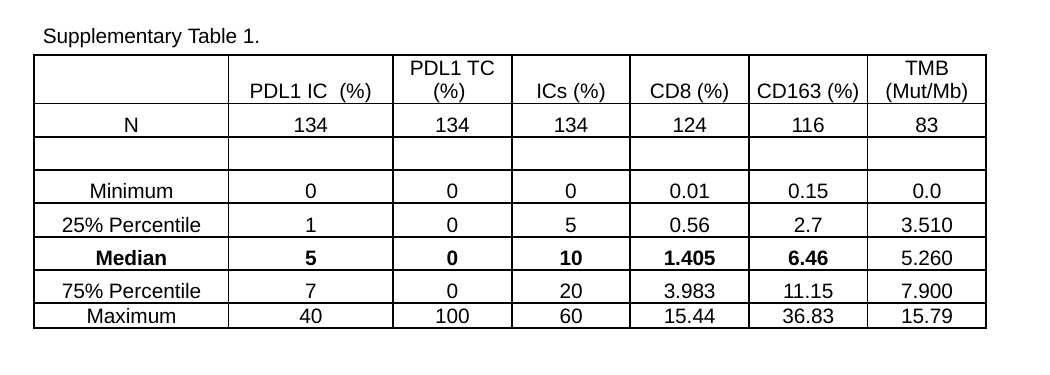

Supplementary Table 1.
| | PDL1 IC (%) | PDL1 TC (%) | ICs (%) | CD8 (%) | CD163 (%) | TMB (Mut/Mb) |
| --- | --- | --- | --- | --- | --- | --- |
| N | 134 | 134 | 134 | 124 | 116 | 83 |
| | | | | | | |
| Minimum | 0 | 0 | 0 | 0.01 | 0.15 | 0.0 |
| 25% Percentile | 1 | 0 | 5 | 0.56 | 2.7 | 3.510 |
| Median | 5 | 0 | 10 | 1.405 | 6.46 | 5.260 |
| 75% Percentile | 7 | 0 | 20 | 3.983 | 11.15 | 7.900 |
| Maximum | 40 | 100 | 60 | 15.44 | 36.83 | 15.79 |
